# Thermal, Mechanical, Chemical, Elemental, Optical and Surface Characterization of Different Twinky Star^®^ Colored Compomers

**DOI:** 10.3390/ma19173663

**Published:** 2026-08-28

**Authors:** Sylwia Klimas, Anna Nikodem, Krzysztof D. Dudek, Agata Małyszek, Maciej Dobrzyński

**Affiliations:** 1 Department of Pediatric Dentistry and Preclinical Dentistry, Wroclaw Medical University, Krakowska 26, 50-425 Wroclaw, Poland; maciej.dobrzynski@umw.edu.pl; 2 Department of Mechanics, Materials and Biomedical Engineering, Faculty of Mechanical Engineering, Wroclaw University of Science and Technology, Ignacego Lukasiewicza 7/9, 50-371 Wroclaw, Poland; krzysztof.dudek@pwr.edu.pl; 3 Department of Biostructure and Animal Physiology, Wrocław University of Environmental and Life Sciences, Kozuchowska 1, 51-631 Wroclaw, Poland; agata.malyszek@upwr.edu.pl

**Keywords:** Twinky Star, dental restorative materials, thermography, light curing, microhardness, SEM, EDS, ATR-FTIR, contact angle, surface free energy

## Abstract

Colored compomers are widely used in pediatric restorative dentistry because their visual attractiveness may improve patient acceptance and cooperation. Although different color variants are often regarded as purely aesthetic modifications, pigments and decorative fillers may influence polymerization, heat generation, mechanical performance, and surface properties. This study evaluated the thermal, mechanical, chemical, elemental, optical, and surface characteristics of four Twinky Star^®^ compomer shades (Blue, Gold, Lemon, and Silver) compared with two commonly used tooth-shaded resin composites, Boston A3 and Charisma A2. Thermographic analysis determined maximum temperature, temperature increase, time above 40 °C, and thermal exposure above 40 °C. Mechanical properties were assessed by instrumented indentation, morphology and elemental composition by SEM/BSE and EDS, chemical characteristics by ATR-FTIR spectroscopy, optical properties by spectrophotometry, and surface characteristics by contact angle measurements. Pronounced differences among the Twinky Star^®^ color variants were observed. Blue showed the lowest thermal response, whereas Lemon exhibited the highest thermal exposure. Gold demonstrated the weakest mechanical performance, while Silver showed the most favorable mechanical profile. EDS revealed quantitative differences in Ba, Sr, F, and Si, whereas ATR-FTIR confirmed a comparable chemical matrix among color variants. These findings indicate that Twinky Star^®^ color variants should not be considered solely aesthetic alternatives, as shade-associated compositional and optical differences may be accompanied by differences in material performance.

## 1. Introduction

The selection of an appropriate restorative material is essential for the long-term success of dental treatment. Restorative materials must exhibit adequate mechanical, chemical, thermal and surface properties to withstand the complex conditions of the oral environment. In light-cured resin-based materials, the final properties depend on both material composition and curing conditions, including exposure time, light intensity, curing distance, material shade, translucency and filler content [1,2,3,4,5,6,7].

Resin-based restorative materials include resin composites and compomers, which differ in composition, setting mechanism and clinical application [8,9,10,11,12,13,14]. Resin composites are based on an organic dimethacrylate matrix and inorganic fillers, and their setting relies primarily on light-induced polymerization [15,16,17,18]. Compomers, also known as polyacid-modified resin composites, combine selected features of composites and glass-ionomer-based materials [19,20,21]. They undergo primarily light-activated polymerization with delayed acid–base reactions after water uptake. In pediatric dentistry, colored compomers such as Twinky Star^®^ may also serve as motivational tools due to their colorful, glitter-like appearance. However, pigments and decorative phases may influence optical absorption, light scattering and polymerization behavior [22,23,24,25].

Light curing is a critical factor affecting the clinical performance of resin-based restorative materials [26,27,28]. Insufficient curing may result in reduced mechanical properties, increased residual monomer content and lower surface hardness [1,28,29]. Conversely, longer or more intense irradiation may improve polymerization, but may also increase temperature rise.

**Figure 1:**
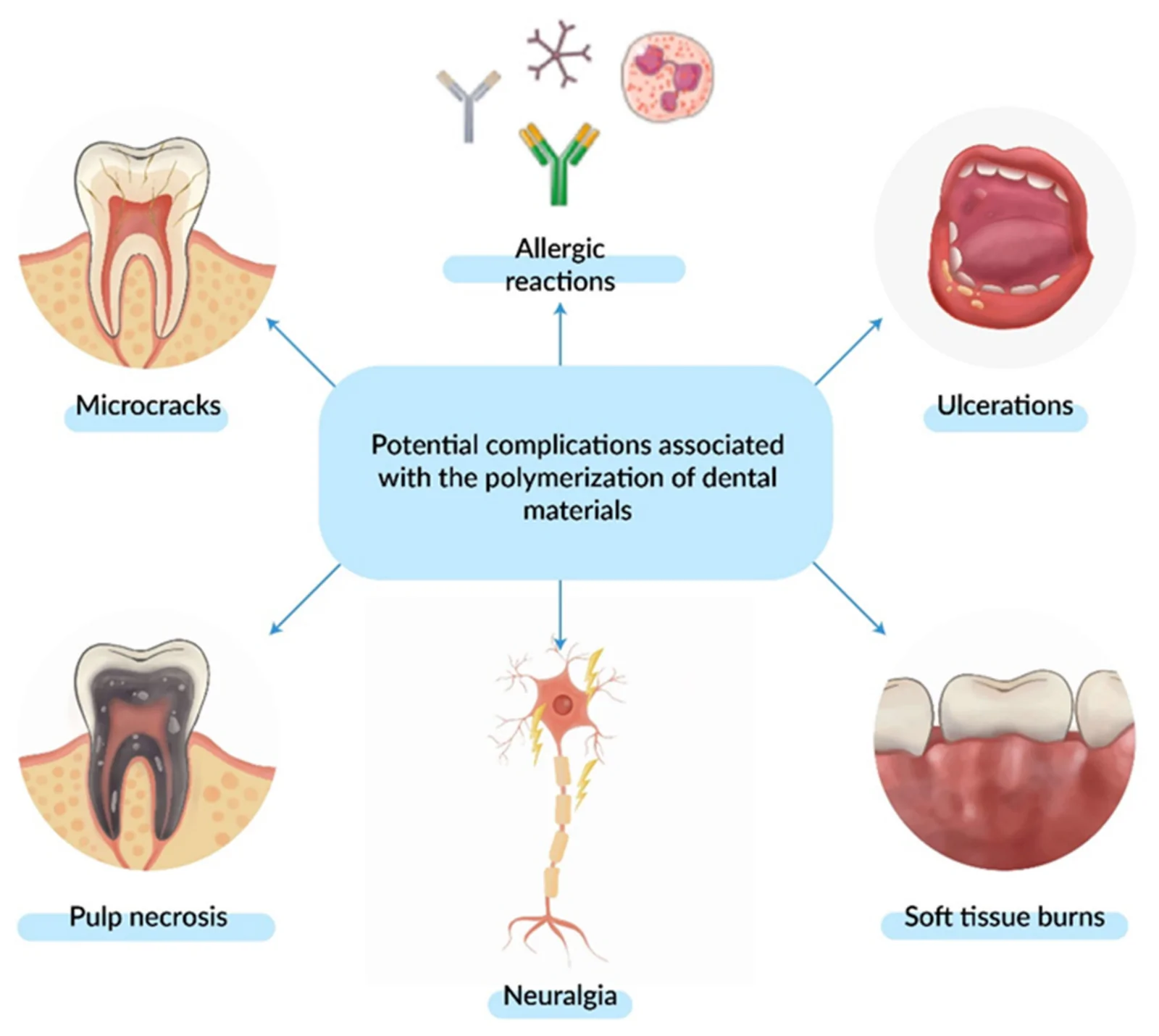
Potential complications associated with the polymerization of dental materials.

Heat generated during light curing results from both the exothermic polymerization reaction and radiant energy from the curing unit. Excessive thermal exposure, particularly near the pulp chamber, remains an important safety consideration, although the commonly cited threshold of a 5.5 °C intrapulpal temperature increase for irreversible pulp damage remains debated [27,30,31,32,33]. Such thermal exposure may contribute to biological and structural complications (Figure 1). Excessive temperature rise may induce pulpal inflammation or necrosis [27,30,31,32,33,34] and adversely affect oral soft tissues [35,36]. Rapid temperature changes may also generate thermal stresses within enamel and dentin [36,37,38,39]. Inadequate polymerization may increase the release of residual monomers associated with hypersensitivity and allergic reactions [40,41,42]. Prolonged thermal exposure may also contribute to irritation of adjacent neural structures and surrounding tissues [33,43,44,45]. These potential effects highlight the importance of controlling thermal exposure during polymerization.

Thermographic analysis provides a non-invasive method for monitoring temperature distribution and temperature–time profiles during polymerization [46,47,48]. Thermal exposure depends on both the magnitude and duration of heating; therefore, time above a defined temperature threshold and the area under the temperature–time curve may provide more comprehensive information than maximum temperature alone. In colored compomers, pigments and decorative phases may further modify light absorption, scattering and heat generation during curing [25,49,50,51]. Thus, different color variants of the same product line should not automatically be assumed to have identical thermal behavior [49,50].

Mechanical properties are another key determinant of restorative material performance. Surface hardness and indentation modulus reflect the resistance of the material to indentation, deformation and potential wear [52,53,54,55,56]. These properties are influenced by the degree of polymerization, filler type, filler content, filler–matrix interaction and curing protocol [25,52,53]. In colored materials, pigments and decorative phases may affect light penetration and polymerization efficiency, potentially influencing mechanical performance [22,50].

Surface characteristics provide further information on material behavior. Wettability and surface free energy influence interactions with water, saliva, proteins, stains and microorganisms [57,58]. Contact angle measurements using polar and non-polar liquids allow estimation of total surface free energy and its polar and dispersive components [59,60]. These measurements provide useful information on surface physicochemical properties, although they do not independently predict long-term degradation [59,61].

Elemental composition and microstructure may also contribute to material behavior. SEM/BSE imaging enables assessment of morphology and filler distribution, whereas EDS provides information on elemental composition [62,63]. Elements such as Si, Al, F, Sr and Ba are commonly associated with glass-based and radiopaque filler systems [8,64]. Differences in elemental composition may occur even when materials exhibit broadly similar morphology [62,65].

The present study focused on four color variants of Twinky Star^®^ colored compomer—Blue, Gold, Lemon and Silver—and included two commonly used tooth-shaded resin composites, Boston A3 and Charisma A2, for comparative purposes. The study combined thermographic analysis during light curing, instrumented indentation testing, SEM/BSE imaging, EDS elemental analysis, ATR-FTIR chemical analysis, spectrophotometric color analysis and contact angle-based surface free energy measurements. This approach aimed to determine whether color variants differ beyond their visual appearance in functional material properties.

The aim of this study was to evaluate the thermal, mechanical, chemical, elemental, optical, and surface characteristics of different Twinky Star^®^ color variants and to determine whether these variants exhibit differences extending beyond their visual appearance. Boston A3 and Charisma A2, two commonly used tooth-shaded resin composites, were included to provide a broader comparative context.

## 2. Materials and Methods

### 2.1. Materials

The study included four color variants of the colored compomer Twinky Star^®^: Blue, Gold, Lemon and Silver. Boston A3 and Charisma A2 were included for comparative purposes. All materials were prepared according to the manufacturers’ instructions (Table 1).

Cylindrical specimens, 6 mm in diameter and 2.5 mm in height, were prepared using a dedicated polytetrafluoroethylene (PTFE) mold. The mold geometry and specimen preparation procedure followed the standardized protocol previously developed and published by the authors [66]. This protocol was used to ensure reproducible specimen dimensions, comparable curing conditions and consistent sample geometry across all investigated materials. The materials were carefully adapted into the mold to minimize air entrapment and ensure uniform specimen geometry before polymerization.

For thermographic measurements, eight independent specimens were prepared and analyzed for each material and curing condition (*n* = 8). For the subsequent mechanical and spectrophotometric characterization, five independent specimens were used per experimental group (*n* = 5). The numbers of measurements used for the remaining characterization procedures are specified in the corresponding methodological subsections. In the instrumented indentation analysis, five indentations were performed on each specimen. The individual indentations were considered technical replicates, whereas the specimen was considered the experimental unit. Therefore, the mean value obtained from the five indentations performed on each specimen was used for subsequent statistical analysis.

### 2.2. Thermographic Measurements

**Figure 2:**
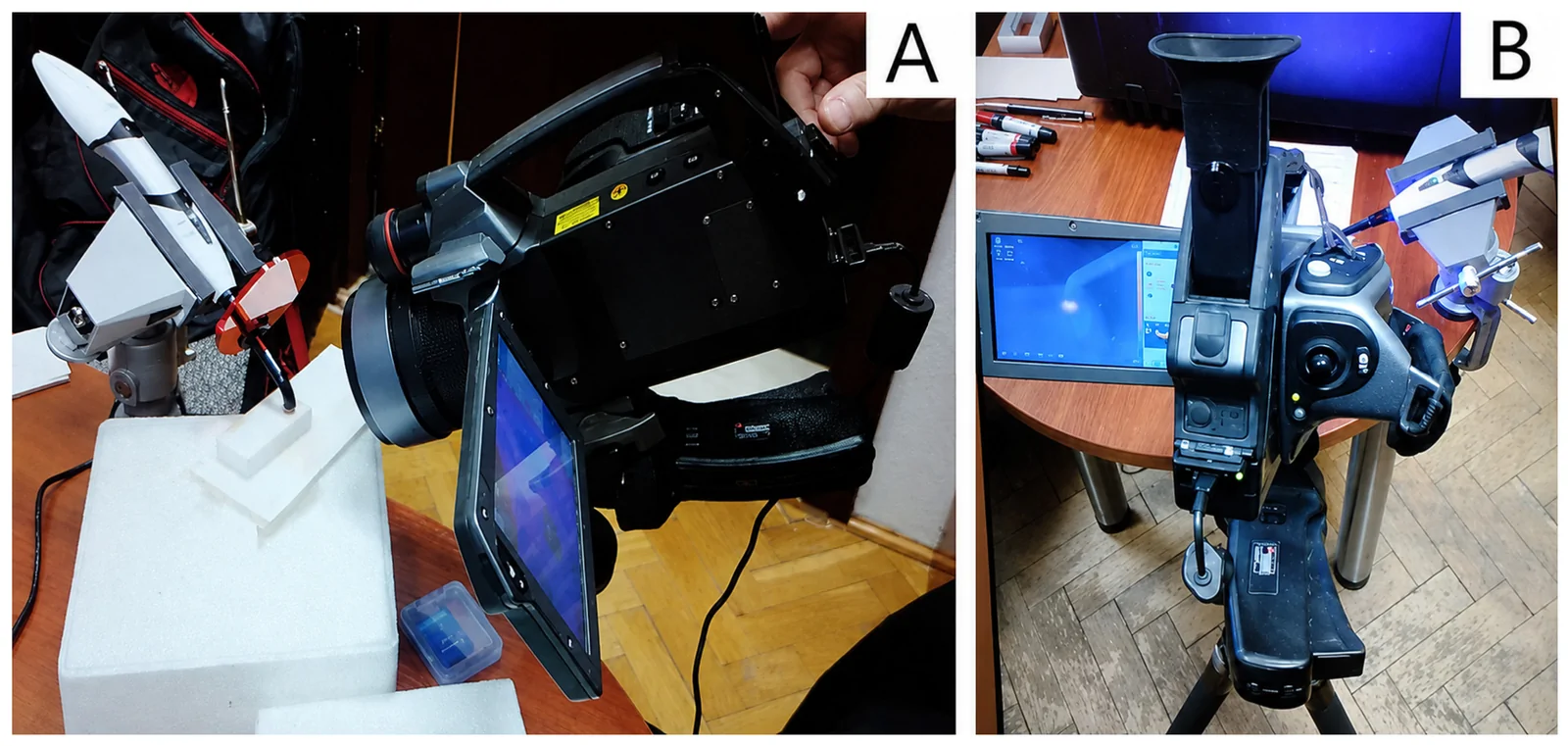
(**A**) Experimental setup for thermographic measurements of dental material samples using a FLIR ThermaCAM P640 thermal imaging camera and a Bluephase Style 20i curing lamp. (**B**) Representative thermographic image recorded during the polymerization process of a material sample.

Thermographic measurements were performed during light curing using a FLIR ThermaCAM P640 thermal imaging camera (FLIR Systems, Goleta, CA, USA). The experimental setup included the dental restorative material specimen, the Bluephase Style 20i curing unit (Ivoclar Vivadent AG, Schaan, Liechtenstein), and the thermal camera positioned to record the temperature distribution on the specimen surface during irradiation. Temperature changes were recorded continuously throughout the polymerization process. The thermal camera was equipped with an uncooled microbolometer detector with a resolution of 640 × 480 pixels and a thermal sensitivity of <0.05 °C at 30 °C, with an operating temperature range from −40 °C to +120 °C (Figure 2). Measurements were performed using a standard lens with a field of view of 30° × 22.5° at a frame rate of 60 Hz. Manual focusing was used to ensure stable image acquisition during the experiment. The curing-light tip was positioned perpendicular (90°) to the specimen surface, with the center of the light guide aligned with the center of the specimen. The distance between the light-guide tip and the specimen surface was maintained at 1.5 mm throughout the main experimental protocol.

The following thermographic parameters were determined: initial temperature at the beginning of irradiation (T0), maximum recorded temperature (Tmax), time to reach maximum temperature (tmax), temperature at the end of light exposure (T1), duration for which the material temperature exceeded 40 °C (t_40°C_), temperature increase (ΔT = Tmax − T0), and the area under the temperature–time curve above 40 °C (A40). The A40 parameter was used as an indicator of accumulated thermal exposure because it integrates both the magnitude and duration of temperature elevation.

For the curing-time protocol, Twinky Star^®^ Blue, Lemon, Gold and Silver, Charisma A2 and Boston A3 specimens were prepared in two experimental sets and light-cured for either 20 s or 40 s. For the curing-distance protocol, additional Boston A3 specimens were light-cured for 20 s at two distances between the curing light tip and the sample surface: 2.5 mm and 7 mm. Eight independent specimens were evaluated for each material and curing protocol (*n* = 8). These protocols were used to compare the effects of material shade, curing time and curing distance on the measured thermal and mechanical properties.

### 2.3. SEM/BSE Imaging and EDS Analysis

Microscopic evaluation was performed using complementary stereomicroscopic and SEM/BSE observations. Prepared specimen surfaces were first examined using a Zeiss SteREO Discovery V20 stereoscopic microscope (Carl Zeiss Microscopy GmbH, Jena, Germany) to verify sample preparation quality, identify visible surface defects and document the shade-dependent surface appearance, including the decorative glitter-like phase in the colored Twinky Star^®^ compomers.

Cross-sectional morphology was then analyzed using a Thermo Scientific™ Phenom Pharos™ G2 Desktop FEG-SEM scanning electron microscope (Thermo Fisher Scientific, Waltham, MA, USA). The specimens were examined in backscattered electron imaging mode (BSE), which provides compositional contrast between phases with different average atomic numbers. This mode was used to assess the internal morphology of polished cross-sections, including filler distribution, filler particle morphology, porosity, cracks, interfacial gaps and structural heterogeneity.

Elemental composition was determined using energy-dispersive X-ray spectroscopy (EDS) integrated with the Thermo Scientific™ Phenom Pharos™ G2 Desktop FEG-SEM system (Thermo Fisher Scientific, Waltham, MA, USA). EDS measurements were performed on representative areas of polished cross-sections of Twinky Star^®^ Blue, Gold, Lemon and Silver, Boston A3 and Charisma A2. For each material, both atomic and weight concentrations were recorded. The interpretation focused mainly on weight concentration, particularly for heavier filler-related elements such as strontium and barium. The EDS analysis was used to compare the elemental profiles of the investigated materials and to identify differences in filler-related elements, including Si, Al, F, P, Sr and Ba.

### 2.4. Contact Angle Measurements and Surface Free Energy Determination

Surface wettability was evaluated using static contact angle measurements. The measurements were performed with an optical contact angle goniometer Surftens Universal (OEG GmbH, Frankfurt am Main, Germany). The method was based on the analysis of the shape of a liquid droplet deposited on the polished specimen surface under controlled conditions.

Two probe liquids with known surface tension parameters were used: distilled water as a polar liquid and diiodomethane as a non-polar liquid. For each measurement, a single droplet of 0.60 ± 0.01 μL was carefully dispensed onto the specimen surface. The contact angle was recorded within 1 s after droplet deposition to minimize the influence of evaporation, absorption or spreading. For each specimen, independent measurements were performed using both test liquids. The number of droplets varied between materials depending on sample availability and is reported together with the results.

Surface free energy was calculated from the contact angle values using the Owens–Wendt approach, which enables separation of the total surface free energy into dispersive and polar components. The following parameters were determined: dispersive component of surface free energy (γs^d^), polar component of surface free energy (γs^p^), total surface free energy (γs total) and Polar Ratio, defined as the percentage contribution of the polar component to the total surface free energy.

### 2.5. Spectrophotometric Color Measurements

The color characteristics of the investigated materials were evaluated using a TS7010 spectrophotometer (3nh Technology Co., Ltd., Shenzhen, China). Measurements were performed in both the CIE L*a*b* and CIE L*C*h° color spaces. The following color parameters were recorded: lightness (L*), chroma (C*), hue angle (h°), and chromatic coordinates (a* and b*).

For each material, five specimens were prepared and analyzed. Each specimen was measured three times, and the obtained values were used to calculate the mean and standard deviation for each color parameter. All measurements were performed under the same experimental conditions to ensure comparability between the tested materials.

### 2.6. ATR-FTIR Spectroscopic Measurements

The chemical structure of the investigated Twinky Star^®^ color variants was analyzed using attenuated total reflectance Fourier-transform infrared spectroscopy (ATR-FTIR). Spectra were recorded using a Thermo Scientific™ Nicolet™ 6700 FTIR spectrometer (Waltham, MA, USA) equipped with a PIKE GladiATR™ ATR accessory. Measurements were performed in absorbance mode in the wavenumber range of 4000–400 cm^−1^. Before sample measurements, a background spectrum was collected and used as the reference for subsequent measurements.

For each Twinky Star^®^ color variant, replicate ATR-FTIR spectra were collected relative to the background spectrum and averaged. The final spectra are presented as mean absorbance values with standard deviation bands. The analysis included Twinky Star^®^ Blue, Gold, Lemon and Silver. For the Silver group, one outlying spectrum was excluded prior to averaging due to its inconsistent course compared with the remaining measurements.

### 2.7. Mechanical Testing

After light curing and thermographic analysis, the dental restorative material specimens were placed in cylindrical silicone molds and embedded in Duracryl^®^ Plus acrylic resin (SpofaDental, Jičín, Czech Republic). The embedded specimens were sectioned to dimensions suitable for mechanical testing using a STRUERS^®^ Accutom-5 metallographic cutter (Struers ApS, Ballerup, Denmark) under water cooling. The specimen surfaces were subsequently wet-polished using 1000-grit abrasive paper to obtain standardized flat surfaces for indentation measurements. Prepared surfaces were inspected using a Zeiss SteREO Discovery V20 stereoscopic microscope to verify preparation quality and exclude visible defects that could affect indentation measurements. Five specimens from each experimental group previously subjected to thermographic analysis were used for subsequent instrumented indentation testing.

**Figure 3:**
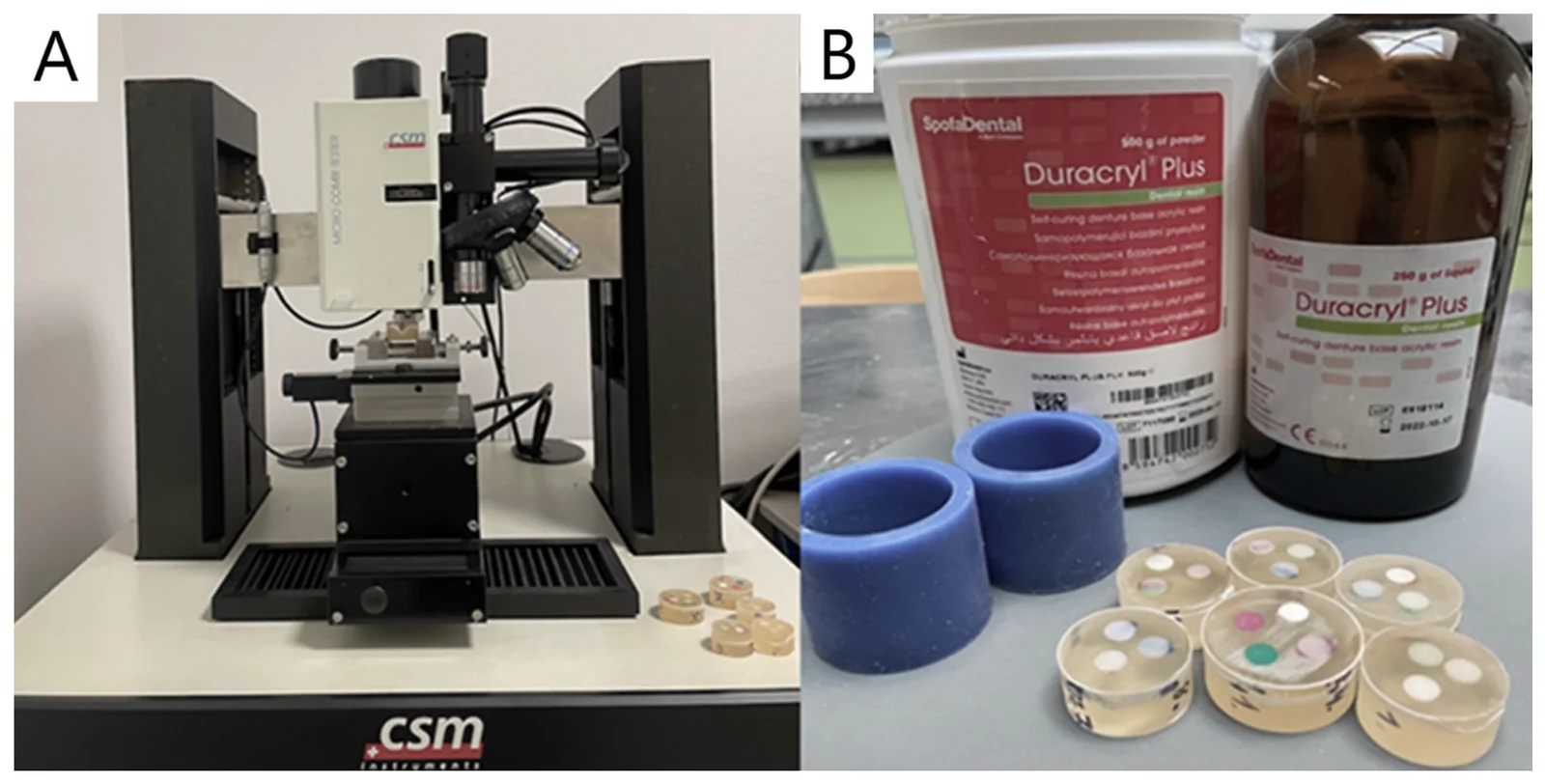
Instrumented indentation testing of dental restorative material specimens: (**A**) CSM MicroCombi Tester™ testing setup; (**B**) specimens embedded in Duracryl® Plus acrylic resin.

Mechanical properties were assessed using instrumented indentation in accordance with ISO 14577 [67]. Measurements were performed with a CSM MicroCombi Tester™ microhardness tester (CSM Instruments SA, Peseux, Switzerland) equipped with a Berkovich indenter with an apex angle of 65.3° and a tip radius below 150 nm (Figure 3). The tests were performed under load-controlled conditions with a maximum load of 400 mN; therefore, indentation depth was a measured response to the applied load rather than a predefined test parameter. The measurements were performed under load-controlled conditions; therefore, indentation depth was a measured response to the applied load rather than a predefined test parameter. Five indentations were performed on each specimen. All measurements were conducted at room temperature, approximately 22 °C, and at a relative humidity of 40–50%.

During each indentation cycle, the load was gradually increased until the maximum force was reached, after which the indenter remained in contact with the specimen for a defined dwell time before unloading. The resulting load–displacement curves were analyzed using Indentation 5.21 software. Mechanical parameters were calculated from the unloading segment of the curve according to the Oliver–Pharr approach, in which the contact stiffness and projected contact area are used to determine hardness and elastic response.

The following parameters were determined: Vickers hardness (HV), Martens hardness (HM), indentation hardness (HIT), indentation modulus (EIT), and the elastic part of indentation work (ηIT). Measurements were performed for Twinky Star^®^ Blue, Gold, Lemon, and Silver after 20 s and 40 s curing. Charisma A2 was also evaluated after 20 s and 40 s curing. Boston A3 was assessed under standard curing conditions and at different curing distances. Five independent specimens were analyzed per experimental group (*n* = 5), with five indentations performed on each specimen.

### 2.8. Data Analysis

Sample size estimation was performed using G*Power, version 3.1.9.7 (Heinrich Heine University Düsseldorf, Düsseldorf, Germany). Based on preliminary pilot observations, a large effect size (Cohen’s f = 0.40) was assumed. The experimental design comprised six materials (four Twinky Star^®^ shades and two commonly used resin composites included for comparison) and two curing times (20 and 40 s), resulting in twelve experimental conditions. For a two-way ANOVA, assuming α = 0.05 and a statistical power of 1 − β = 0.80, the estimated minimum total sample size was 76–84 observations, corresponding to approximately seven observations per experimental condition. Therefore, eight observations per group were included in the study.

Results are presented as mean ± standard deviation (SD). Statistical analyses were selected according to the structure of the individual datasets. For the thermographic analysis, the effects of material type and curing time on the maximum temperature (Tmax) were evaluated using two-way ANOVA, with material and curing time as fixed factors. The Material × Curing time interaction was included in the model to determine whether the effect of prolonged light exposure depended on the investigated material.

For comparisons among the four Twinky Star^®^ color variants in the spectrophotometric and wettability analyses, Welch’s one-way ANOVA followed by the Games–Howell post hoc test was used where appropriate. Boston A3 and Charisma A2 were included as commonly used tooth-shaded resin composites for comparative purposes and were not included in statistical analyses specifically intended to assess differences among the Twinky Star^®^ color variants.

For the instrumented indentation parameters of the Twinky Star^®^ materials, the effects of shade and curing time were evaluated using two-way ANOVA, including the Shade × Curing time interaction. Effect sizes were expressed as partial eta squared (partial η^2^). For Charisma A2, differences between the 20 and 40 s curing conditions were evaluated using Welch’s *t*-test, with Hedges’ g reported as the effect size. The Boston A3 experimental conditions were compared using Welch’s one-way ANOVA followed by the Games–Howell post hoc test where appropriate.

Differences between selected experimental conditions were additionally expressed as absolute and percentage differences, where appropriate. Statistical significance was set at *p* < 0.05.

## 3. Results

### 3.1. Thermographic Analysis of Dental Materials

**Figure 4:**
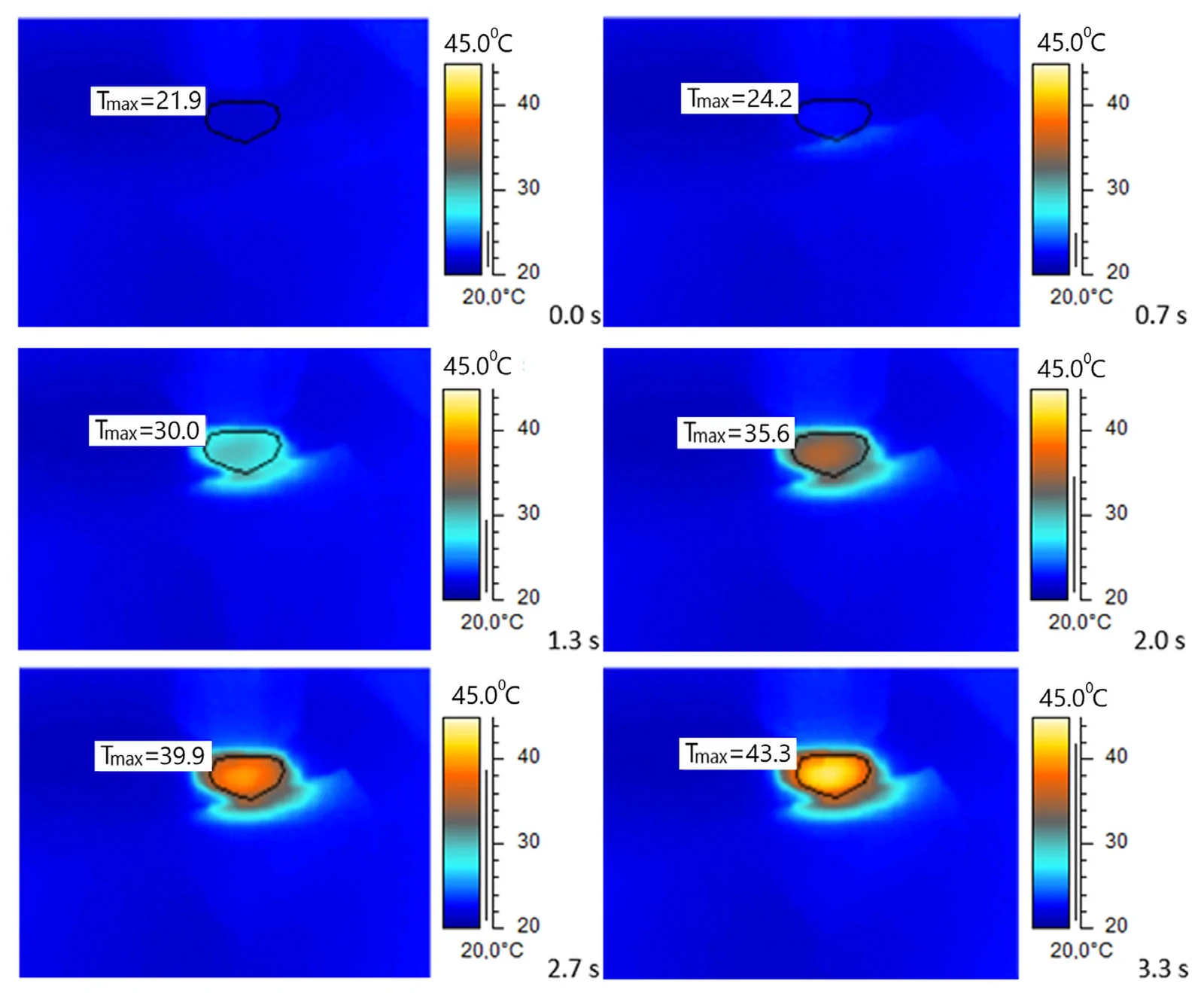
Representative thermographic heatmaps showing temperature distribution on the surface of a dental restorative material sample at selected time points during light curing.

The filling samples were examined during polymerization using a FLIR ThermaCAM P640 thermal imaging camera. Representative thermographic heatmaps were recorded to visualize the evolution of temperature distribution on the sample surface during light curing (Figure 4). The analyzed materials included four Twinky Star^®^ compomer shades—Blue, Gold, Lemon and Silver—as well as Boston A3 and Charisma A2 composite materials.

**Figure 5:**
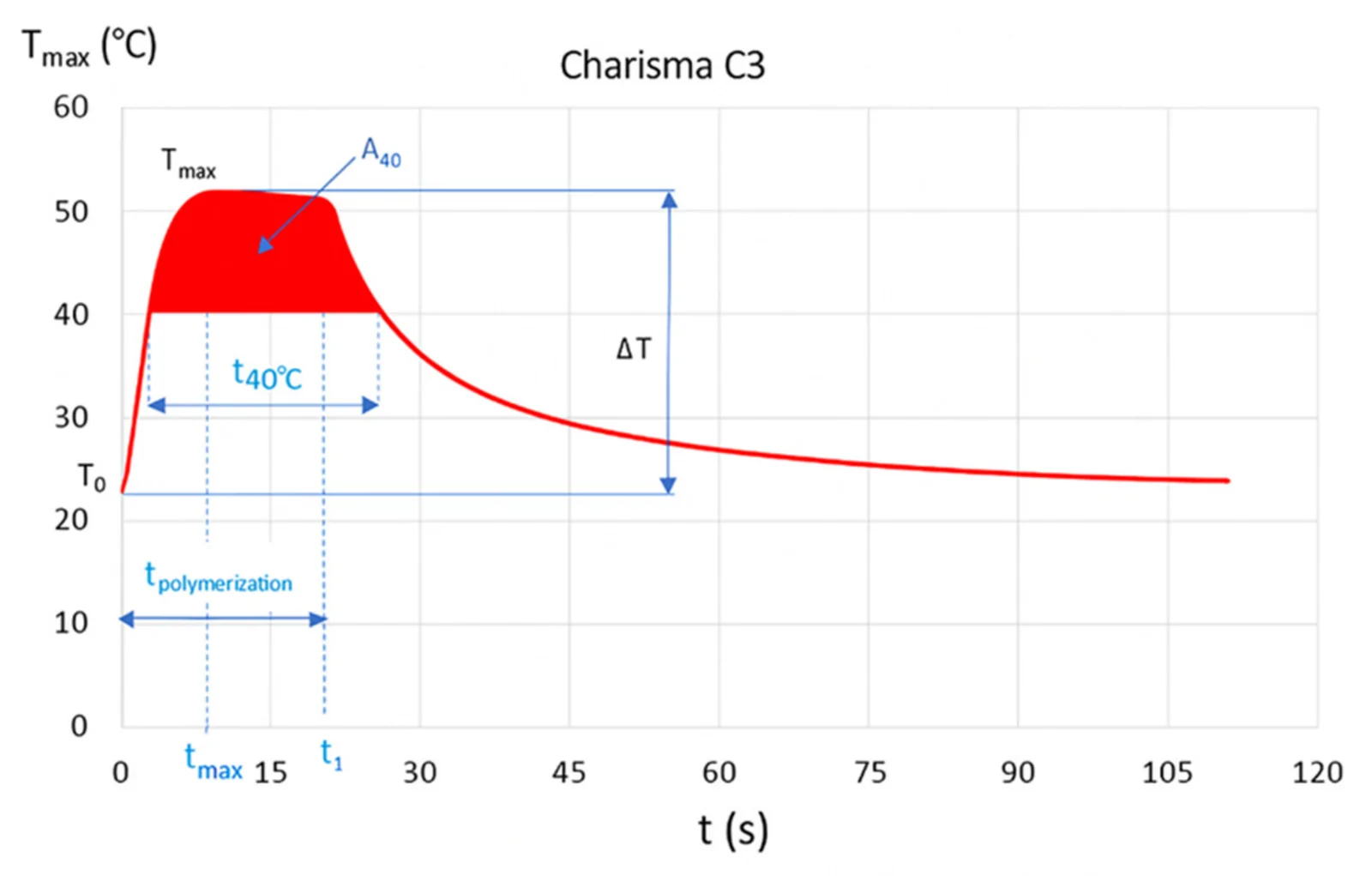
Representative temperature–time profile recorded during light curing of a restorative material sample. The graph illustrates the thermographic parameters used for quantitative analysis: T0, initial temperature; Tmax, maximum temperature; tmax, time to reach maximum temperature; T1, temperature at the end of light exposure; ΔT, temperature increase; t_40°C_, duration above 40 °C; and A40, area under the temperature–time curve above 40 °C.

For quantitative analysis, temperature–time profiles were generated for each material and curing protocol, the temperature characteristics of the Twinky Star® materials are presented in Table 2, whereas those of Boston A3 and Charisma A2 are presented in Table 3. Figure 5 presents a representative curve with the main parameters used in the analysis: initial temperature (T0), maximum temperature (Tmax), time to reach maximum temperature (tmax), temperature at the end of light exposure (T1), temperature increase (ΔT = Tmax − T0), duration above 40 °C (t_40°C_) and the area under the temperature–time curve above 40 °C (A40). The 40 °C threshold was used to compare elevated thermal exposure between materials. Because the initial temperature was comparable for all samples, approximately 22.5 °C, the analysis focused mainly on Tmax, t_40°C_, ΔT and A40.

The two-way ANOVA results for the effects of material type and curing time on maximum polymerization temperature are presented in Table 4.

**Figure 6:**
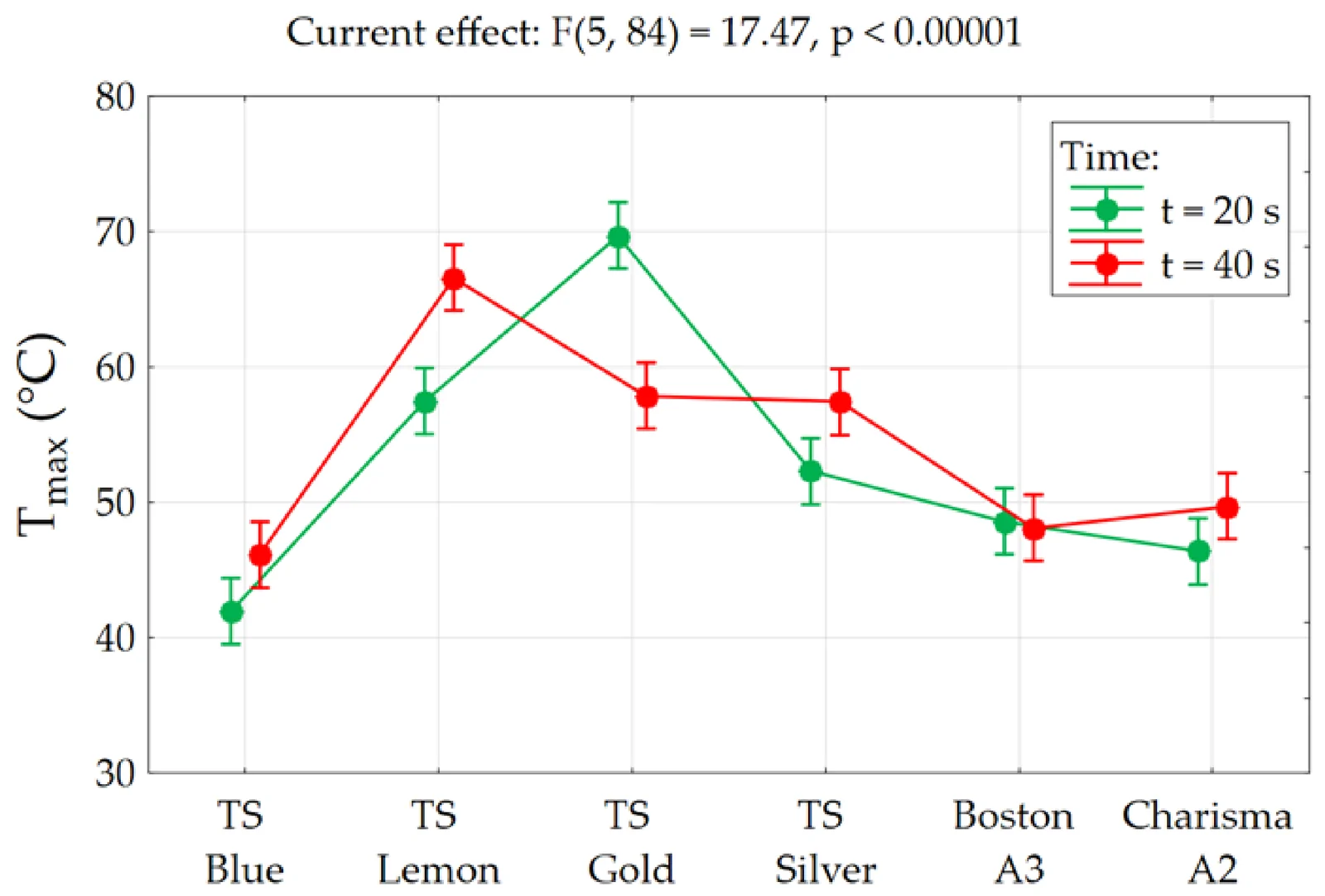
Interaction between material type and curing time (20 and 40 s) for the maximum polymerization temperature (Tmax). Points represent mean values, and vertical bars indicate 95% confidence intervals. Two-way ANOVA revealed a significant material × curing time interaction (*F*(5,84) = 17.47, *p* < 0.001), indicating that the effect of curing time on Tmax depended on the material tested.

The interaction between material type and curing time for maximum polymerization temperature (*T*_max_) is shown in Figure 6.

The significant interaction reflected distinct material-dependent responses to prolonged curing. Twinky Star^®^ Lemon showed a pronounced increase in Tmax after extending the curing time from 20 to 40 s, whereas Twinky Star^®^ Gold showed a decrease in Tmax. In contrast, the changes observed for Twinky Star^®^ Blue, Boston A3, and Charisma A2 were comparatively smaller. Thus, the effect of curing time on maximum temperature cannot be interpreted independently of material type.

All investigated materials exceeded the analytical threshold of 40 °C during light curing; however, the magnitude and duration of thermal exposure differed markedly between groups. Among the Twinky Star^®^ variants, Blue showed the lowest thermal response. Although Tmax, t_40°C_, and A40 increased after 40 s curing, Blue remained the least thermally active material.

Twinky Star^®^ Lemon exhibited the highest accumulated thermal exposure among all tested materials. After 40 s curing, Lemon reached the highest A40 value, indicating the greatest cumulative thermal load. Compared with Blue cured for 40 s, Lemon showed an A40 value higher by 2624 s·°C, corresponding to a 256.3% increase. Twinky Star^®^ Gold also showed a strong thermal response, particularly after 20 s curing, when it reached the highest Tmax among the Twinky Star^®^ variants. However, after 40 s curing, Tmax decreased, while t_40°C_ and A40 increased, indicating that prolonged curing increased the duration of elevated temperature rather than the peak temperature. Silver showed an intermediate thermal profile within the Twinky Star^®^ group.

Boston A3 and Charisma A2 showed moderate thermal behavior under the investigated curing conditions. For Boston A3, increasing curing time from 20 s to 40 s did not increase Tmax, which remained nearly unchanged; however, t_40°C_ and A40 increased markedly. Similarly, Charisma A2 showed higher t_40°C_ and A40 values after 40 s curing. The Boston A3 specimens tested at different curing distances showed considerable variability, particularly at 7 mm, where the high standard deviation suggests less stable thermal behavior under increased distance from the curing light.

Overall, the thermographic results demonstrated that the thermal response of the tested materials depended on both material type and curing protocol. Twinky Star^®^ Lemon generated the highest cumulative thermal exposure, Blue showed the lowest thermal response, and Gold produced high peak temperatures. These findings indicate that maximum temperature alone is insufficient to characterize curing-related heat generation; time-dependent parameters such as t_40°C_ and A40 provide a more comprehensive assessment of thermal exposure during light curing.

### 3.2. Microscopic Evaluation of Sample Surfaces

**Figure 7:**
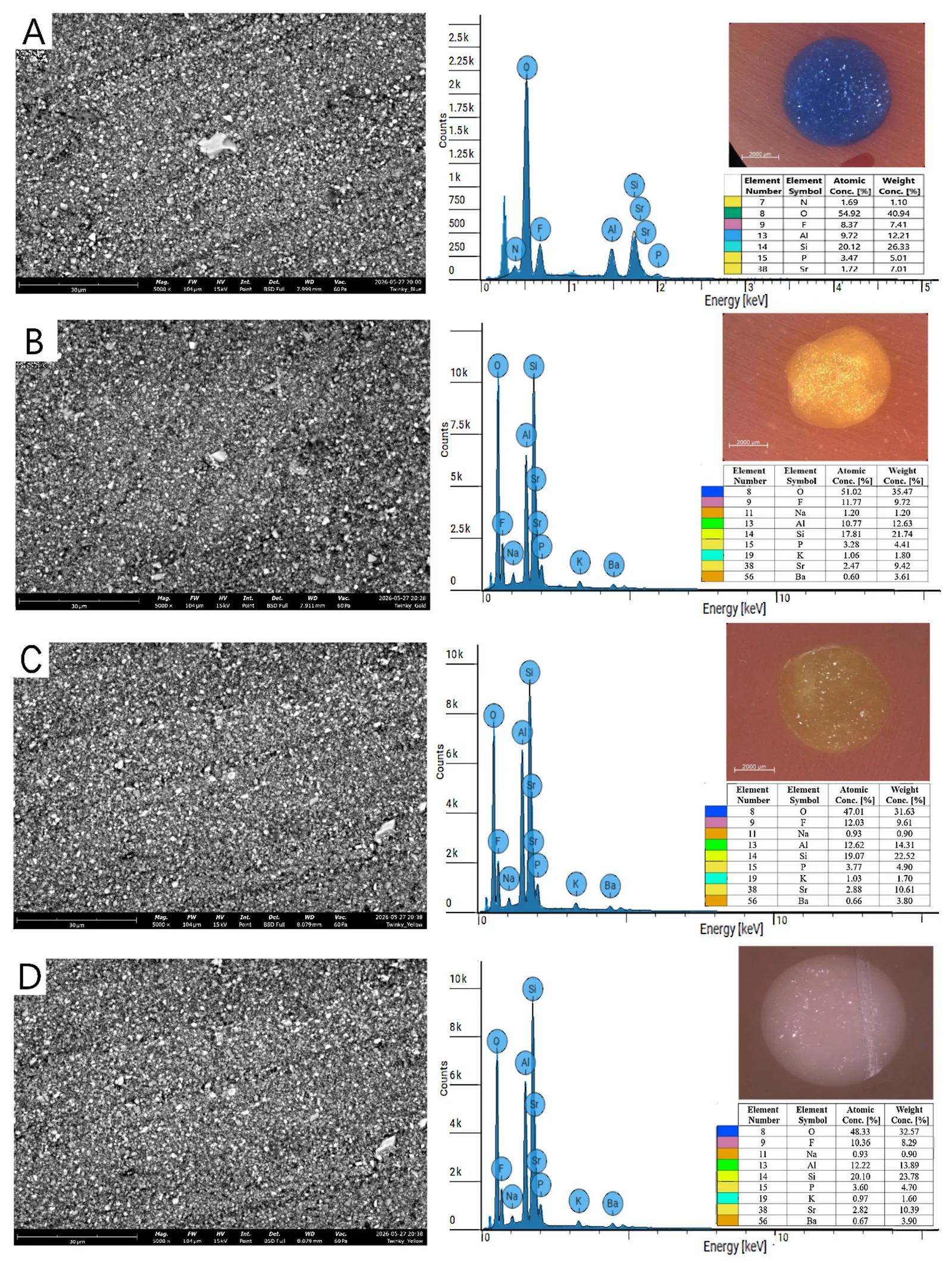
Representative microscopic images of the investigated restorative material samples obtained using a Stereo Discovery V20 stereoscopic microscope: (**A**) Twinky Star^®^ blue compomer, (**B**) Twinky Star^®^ gold compomer, (**C**) Twinky Star^®^ lemon compomer, and (**D**) Boston A3 composite.

**Figure 8:**
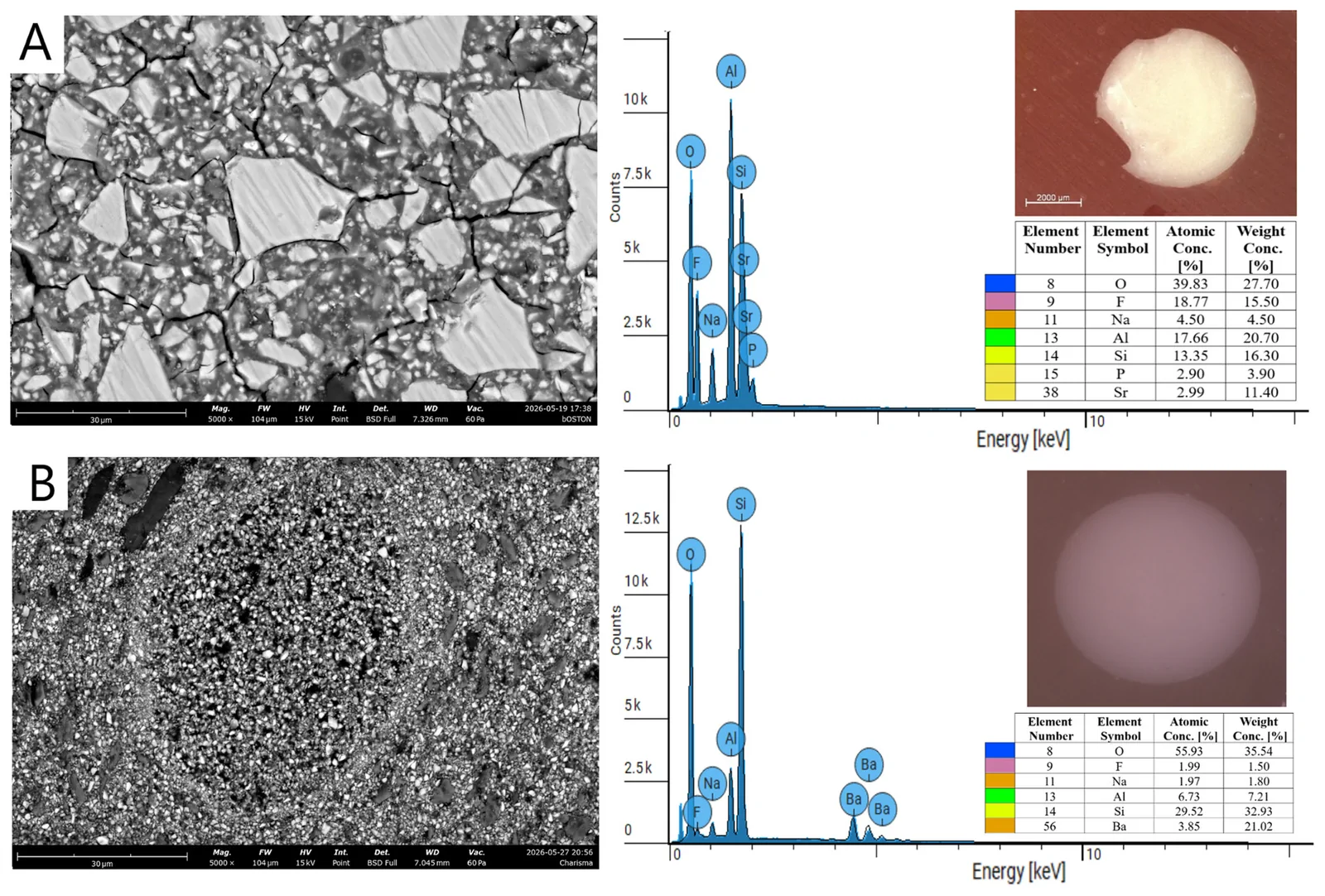
Comparison of the surface morphology, elemental composition, and macroscopic appearance of Boston A3 and Charisma A2 dental materials. Representative SEM images, corresponding EDS spectra, and stereomicroscope images are shown for Boston A3 (**A**) and Charisma A2 (**B**). The images illustrate differences in surface structure, filler distribution, and visual appearance between the two materials.

Microscopic images were obtained using the Zeiss SteREO Discovery V20 microscope to assess the surface appearance of the investigated materials (Figure 7 and Figure 8). SEM analysis revealed heterogeneous surface morphology in all investigated Twinky Star materials, with numerous dispersed particles embedded within the resin matrix. The surfaces showed a granular structure, consistent with the presence of inorganic filler particles. No major surface defects or cracks were observed in the representative images; however, local variations in particle distribution were visible between the different color variants.

EDS spectra confirmed a broadly similar elemental composition among the tested materials. Carbon and oxygen were detected in all samples and were associated mainly with the organic resin matrix. Silicon, aluminum, and barium were also present, indicating the contribution of inorganic glass or silica-based fillers. Additional elements, including sodium, phosphorus, potassium, fluorine, and minor color-related components, were detected depending on the material variant. These differences suggest variations in filler composition and/or pigment additives between the blue, gold, lemon, and silver materials.

Stereomicroscope images showed clear macroscopic differences between the materials, corresponding to their commercial color variants. The blue, gold, lemon, and silver specimens exhibited distinct coloration and visible reflective particles. These decorative particles were distributed throughout the material surface and contributed to the characteristic optical appearance of the tested materials. Overall, the SEM, EDS, and stereomicroscope observations confirmed that the Twinky Star materials share a similar composite structure but differ in color appearance and selected elemental components.

In contrast to the Twinky Star materials, the SEM images of Boston A3 and Charisma A2 revealed different surface morphologies. Boston A3 showed a more irregular and cracked surface, with large plate-like particles embedded in the matrix. The EDS spectrum of Boston A3 confirmed the presence of oxygen, fluorine, sodium, aluminum, silicon, phosphorus, and strontium, indicating a filler system containing aluminosilicate and strontium-containing components.

Charisma A2 exhibited a more homogeneous surface morphology, with numerous fine particles distributed within the resin matrix and without the large plate-like structures observed in Boston A3. The EDS spectrum of Charisma A2 showed mainly oxygen, silicon, aluminum, barium, sodium, and fluorine. The high silicon and barium contents indicate the presence of silica and barium containing glass fillers. Stereomicroscope images confirmed the different macroscopic appearance of both materials: Boston A3 appeared as a light, opaque material with an irregular specimen outline, whereas Charisma A2 showed a smoother, more uniform tooth-colored appearance.

Overall, the SEM and EDS results demonstrated clear differences between the investigated materials. The Twinky Star materials presented a heterogeneous surface with decorative reflective particles and color-dependent elemental variations, while Boston A3 and Charisma A2 showed more conventional restorative composite characteristics, with inorganic filler particles embedded in the resin matrix. Among them, Boston A3 displayed the most irregular surface morphology, whereas Charisma A2 showed a more uniform microstructure.

### 3.3. ATR-FTIR Analysis

**Figure 9:**
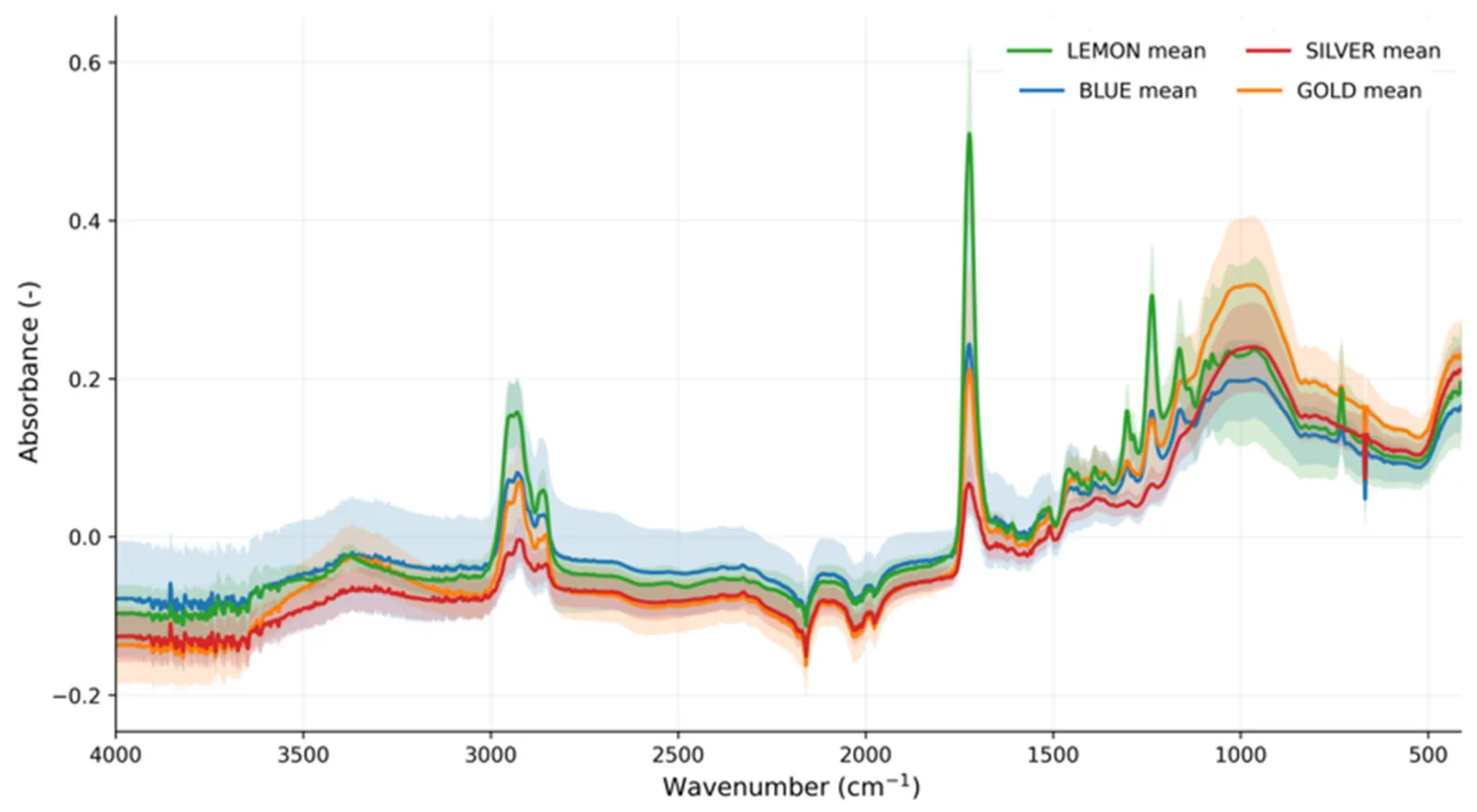
Mean ATR-FTIR spectra of Twinky Star^®^ color variants recorded in absorbance mode relative to the background spectrum. Curves are presented as mean values with standard deviation bands. The Silver group was calculated after exclusion of one outlying spectrum.

ATR-FTIR analysis was performed to compare the chemical profiles of the Twinky Star^®^ color variants (Figure 9). The spectra showed a generally similar course for all investigated colors, indicating that the materials share a comparable resin matrix and filler-related chemical structure. The main differences between the color variants were associated primarily with band intensity rather than with the appearance of entirely new absorption regions.

All spectra showed characteristic bands corresponding to the organic resin matrix and inorganic filler system. Bands in the high-wavenumber region were associated with C–H stretching vibrations of the polymeric matrix, while the intense features in the fingerprint region reflected contributions from ester groups, C–O/C–C vibrations and filler-related Si–O bonds. The presence of similar bands in all color variants indicates that the basic chemical structure of the Twinky Star^®^ materials was preserved regardless of color.

Despite the overall similarity, differences in band intensity were observed between the color variants. These differences were most pronounced in selected regions of the fingerprint range and may be related to variations in pigment systems, decorative particles and filler distribution. Lemon showed the most intense response in selected spectral regions, whereas Silver exhibited the lowest overall intensity in several parts of the spectrum. Blue and Gold showed intermediate behavior, with broadly similar spectral profiles but local differences in band intensity.

Overall, ATR-FTIR confirmed that the investigated Twinky Star^®^ color variants differed mainly in the intensity of selected absorption bands rather than in the presence of distinct chemical groups. This indicates that color modification affected the spectral response of the material, while the general chemical structure remained comparable between the tested variants.

### 3.4. Spectrophotometric Color Analysis

Spectrophotometric analysis demonstrated pronounced differences in color parameters among the investigated materials (Table 5). Boston A3 showed the highest lightness, whereas Twinky Star^®^ Blue exhibited the lowest L values. Among the Twinky Star^®^ variants, Gold showed the highest chroma, followed by Blue and Lemon, whereas Silver exhibited markedly lower color saturation. Twinky Star^®^ Blue was characterized by strongly negative b values, confirming the dominance of the blue component. In contrast, Gold and Lemon showed positive b values, indicating a yellow color component, with Gold additionally showing positive a values. Silver exhibited low chroma and relatively low a and b values, corresponding to a less saturated and more neutral color appearance. Charisma A2 similarly showed low chroma and low absolute values of the chromatic coordinates.

Statistical analysis confirmed significant differences among the four Twinky Star^®^ color variants for all investigated color parameters. Welch’s one-way ANOVA showed significant between group differences for L, C, h°, a, and b (*p* < 0.05 for all parameters). Post hoc Games–Howell analysis showed that Blue had significantly lower L values than Gold, Lemon, and Silver, whereas no significant differences in lightness were found among Gold, Lemon, and Silver. Silver exhibited significantly lower chroma than the other Twinky Star^®^ variants, while no significant differences in C were observed among Blue, Gold, and Lemon.

The hue angle also differed significantly among the Twinky Star^®^ variants. Blue showed a significantly different h° compared with the other shades, and a significant difference was also observed between Gold and Lemon. Significant differences were additionally found for the chromatic coordinates a and b. For b, Blue differed significantly from Gold, Lemon, and Silver, while Silver also differed significantly from Gold and Lemon. Gold and Lemon did not differ significantly in b. These results confirm distinct spectrophotometric characteristics of the individual Twinky Star^®^ color variants. The relatively high variability observed for Silver, particularly in h°, may be associated with the presence of reflective decorative particles and local optical heterogeneity of the material surface. Boston A3 and Charisma A2 were included for descriptive comparison and were not included in the inferential statistical analysis.

### 3.5. Wettability and Surface Free Energy Analysis

Contact angle measurements revealed differences in wettability between the investigated materials. The calculated surface free energy parameters, including the dispersive and polar components, total surface free energy, and polar ratio, are summarized in Table 6. These parameters were used to compare the surface physicochemical characteristics of the tested materials.

Among the Twinky Star^®^ variants, no statistically significant differences were observed in the water contact angle (Welch’s one-way ANOVA, *p* > 0.05), with mean values ranging from 61.23° for Silver to 63.99° for Lemon. In contrast, significant differences were found for the diiodomethane contact angle (*p* < 0.001). Games–Howell post hoc analysis showed significantly higher values for Gold than for Lemon and Blue, while Silver also exhibited a significantly higher value than Blue. The remaining pairwise differences were not statistically significant.

The calculated surface free energy parameters also showed descriptive differences among the investigated materials. Twinky Star^®^ Blue exhibited the highest total surface free energy (53.85 ± 3.40 mJ/m^2^), followed closely by Charisma A2 (53.47 ± 0.64 mJ/m^2^), whereas Boston A3 showed the lowest value (35.71 ± 0.37 mJ/m^2^). Boston A3 also exhibited the highest water contact angle and the lowest polar component and polar ratio, indicating the most hydrophobic and least polar surface among the tested materials. Among the Twinky Star^®^ variants, Gold showed the highest polar ratio (23.92 ± 3.64%), followed by Silver (22.37 ± 4.02%). The two excluded water contact angle values recorded for Silver (>100°) indicate local variability of the surface response; however, these values were not included in the calculation of the final surface parameters.

Overall, the results indicate material-dependent differences in surface wettability and surface free energy. The observed variations may be associated with differences in filler composition, pigmentation, decorative particles, and local surface characteristics.

### 3.6. Mechanical Properties of Materials

Mechanical characterization was performed using instrumented indentation to evaluate the effect of material type, curing time, and, for Boston A3, curing distance on the mechanical response of the tested materials. The analysis included residual indentation depth (hr), Vickers hardness (HV), Martens hardness (HM), indentation hardness (HIT), indentation modulus (EIT), and elastic indentation work ratio (ηIT). The results are presented as mean ± standard deviation (SD) in Table 7 and Table 8.

Two-way ANOVA demonstrated a statistically significant effect of Twinky Star^®^ shade on all investigated indentation parameters (Table 9). The strongest shade effect was observed for HM (partial η^2^ = 0.695), followed by HV and HIT (partial η^2^ = 0.603), hr (partial η^2^ = 0.574), and EIT (partial η^2^ = 0.469). A significant, although smaller, shade effect was also observed for ηIT (partial η^2^ = 0.306).

Curing time had a significant main effect on HM (F(1,24) = 6.84, *p* = 0.015, partial η^2^ = 0.222) and EIT (F(1,24) = 13.31, *p* = 0.001, partial η^2^ = 0.357). No significant main effect of curing time was found for hr, HV, HIT, or ηIT (*p* > 0.05). Furthermore, no significant shade × curing time interaction was observed for any of the investigated indentation parameters (*p* > 0.05), indicating that the effect of curing time did not differ significantly among the four Twinky Star^®^ shades.

Descriptively, Twinky Star^®^ Gold showed the lowest hardness and indentation modulus values, particularly after 20 s of curing, whereas Lemon and Silver generally exhibited higher hardness related parameters. EIT increased from 20 to 40 s in all four Twinky Star^®^ variants, consistent with the significant main effect of curing time identified by the two-way ANOVA.

For Charisma A2, extending the curing time from 20 to 40 s resulted in significantly lower hr and significantly higher HV, HM, HIT, and EIT values (Welch’s *t*-test, *p* < 0.05), whereas the difference in ηIT was not statistically significant (*p* = 0.167). The corresponding effect sizes were large for the hardness- and modulus-related parameters, with Hedges’ g ranging in absolute magnitude from 2.40 to 3.30.

For Boston A3, Welch’s one-way ANOVA indicated significant differences among the investigated curing conditions for HV (*p* = 0.046), HM (*p* = 0.021), HIT (*p* = 0.046), and EIT (*p* = 0.011), whereas differences in hr (*p* = 0.074) and ηIT (*p* = 0.052) did not reach statistical significance. Games–Howell post hoc comparisons identified significant differences between the standard 20 s condition and the 2.5 mm condition for HV, HM, HIT, and EIT. The 7 mm condition showed lower mean hardness and modulus values and markedly greater variability; however, pairwise comparisons involving this condition did not reach statistical significance.

Overall, the instrumented indentation results demonstrated that the mechanical response was strongly dependent on the Twinky Star^®^ shade, whereas the effect of curing time was parameter specific. Among the Twinky Star^®^ variants, curing time significantly affected HM and EIT, while no significant shade × curing time interactions were observed. Charisma A2 showed significant changes in most indentation parameters after prolonged curing, whereas the Boston A3 results indicated condition-dependent differences accompanied by particularly high variability at the 7 mm curing distance.

## 4. Discussion

Polymerization is a key process influencing the performance, durability, and biocompatibility of dental restorative materials, as it directly affects their mechanical properties, wear resistance, and clinical longevity. Inadequate polymerization may compromise restoration quality and patient safety, highlighting the importance of optimizing curing parameters such as light intensity, exposure time, and material thickness [68,69,70]. In recent years, thermographic analysis has gained increasing attention as a tool for evaluating the thermal behavior of dental materials under clinical conditions, particularly during light-curing procedures, where temperature changes may affect both the material and surrounding tissues. Therefore, understanding the relationship between polymerization conditions, thermal effects, and material properties is essential for improving the safety and effectiveness of restorative treatments [71,72,73]. In this context, the present study evaluates the thermal behavior of restorative materials and the influence of curing parameters on their mechanical properties.

The present study demonstrated that different color variants of the same pediatric restorative material may differ in several functional characteristics in addition to their visual appearance. Significant shade-related differences were identified in the instrumented indentation and spectrophotometric analyses, whereas the thermographic analysis demonstrated a strong material effect and a significant material × curing time interaction for Tmax. Differences were also observed in elemental composition and surface physicochemical characteristics. Taken together, these results indicate that the individual Twinky Star^®^ variants cannot necessarily be regarded as functionally identical materials. This observation is consistent with the findings of Hwang et al. [74], who demonstrated marked differences in optical, mechanical, and chemical properties among various Twinky Star^®^ shades despite their common commercial classification. Further evidence was provided by Khodadadi et al. [75], who observed substantial shade-dependent variations in surface hardness, indicating that individual color variants cannot be regarded as functionally identical restorative materials. More recently, Dorterler et al. [50] confirmed that both degree of conversion and microhardness varied significantly among different colored compomers, supporting the concept that pigment systems may influence clinically relevant material properties.

The thermographic analysis confirmed a strong effect of material type on Tmax, together with a smaller but significant effect of curing time. Importantly, the significant material × curing time interaction demonstrated that the effect of extending curing from 20 to 40 s was not uniform across the investigated materials. This was particularly evident for the Twinky Star^®^ variants: Lemon showed an increase in Tmax with prolonged curing, whereas Gold showed the opposite response. Twinky Star^®^ Blue showed the lowest thermal response, whereas Twinky Star^®^ Lemon and Gold generated substantially higher thermal loads. Lemon, particularly after 40 s curing, exhibited the highest accumulated thermal exposure, expressed by the A40 parameter. This indicates that maximum temperature alone is not sufficient to describe the thermal behavior of light-cured restorative materials. Although Tmax provides information about peak temperature, parameters such as t_40°C_ and A40 better reflect the duration and cumulative intensity of heating and may therefore offer a more clinically relevant assessment of thermal exposure. Nevertheless, the shade-dependent differences observed in the present study are consistent with previous investigations evaluating temperature changes during polymerization of colored compomers. Ertuğrul et al. [49] reported significantly greater intrapulpal temperature increases for gold colored materials compared with blue colored variants, while Altan et al. [76] demonstrated that restorative material color significantly affected thermal behavior during curing procedures. These findings collectively support the interpretation that differences in pigmentation, optical properties, decorative phases, and filler-related composition may contribute to variations in heat generation and heat transfer during polymerization.

The high thermal response observed for Lemon and Gold may be related to differences in pigmentation, optical absorption, translucency, and light scattering within the material. Colored and glitter-containing compomers contain decorative particles and pigment systems that can modify the interaction between the curing light and the material. Depending on the shade, these components may absorb or scatter light differently, resulting in differences in heat generation and polymerization dynamics. The fact that Blue showed the lowest thermal response suggests that not all intense colorants increase thermal exposure to the same extent. Instead, the thermal effect appears to depend on the specific optical and compositional characteristics of each color variant. Differences between shades have also been reported with respect to other thermal properties. Guler et al. [77] investigated the thermal conductivity of colored compomers and found significant differences among individual color variants, with the highest thermal conductivity observed for the silver shade and the lowest for the berry shade.

The effect of curing time was also material dependent. In many groups, extending curing time from 20 s to 40 s increased the accumulated thermal exposure, even when Tmax did not increase proportionally. This was particularly evident for Boston A3, where Tmax remained relatively stable between 20 s and 40 s, whereas t_40°C_ and A40 increased markedly. These results indicate that prolonged curing may increase the total thermal burden primarily by extending the duration of elevated temperature, even when Tmax does not increase proportionally. From a clinical perspective, this distinction is important because biological tissues are affected not only by the maximum temperature reached but also by the time for which elevated temperature is maintained. The influence of curing conditions on thermal behavior has been reported in previous studies. Hubbezoglu et al. [78] demonstrated that both the curing mode and restorative material significantly affected temperature rise beneath dentin during composite polymerization. More specifically, studies investigating irradiation time have shown that prolonged exposure may increase thermal development. Duratbegović et al. [79] reported that longer exposure times generally resulted in higher temperature rises in resin-based composites, while Szalewski et al. [45] observed greater temperature increases following extended polymerization protocols compared with shortened curing modes.

Instrumented indentation analysis demonstrated a pronounced shade-dependent mechanical response among the Twinky Star^®^ variants. Two-way ANOVA showed a significant main effect of shade on all investigated indentation parameters, with particularly large effects for HM, HV, HIT, and hr. In contrast, the effect of curing time was parameter specific: a significant main effect was observed for HM and EIT, whereas hr, HV, HIT, and ηIT did not show a significant overall effect of curing time. No significant shade × curing time interaction was identified for any of the mechanical parameters. Thus, although the mean values of several hardness related parameters were higher after 40 s curing, the statistical results indicate that the influence of prolonged curing was more clearly reflected in Martens hardness and indentation modulus than in the remaining indentation parameters.

Among the Twinky Star^®^ variants, Gold showed the lowest hardness and modulus related values, particularly after 20 s curing, whereas Lemon and Silver generally exhibited higher values. Gold is especially noteworthy because it combined a high thermal response with comparatively lower mechanical performance. This indicates that greater heat generation during polymerization does not necessarily correspond to greater hardness or stiffness. Previous studies have linked the mechanical properties of resin-based materials to polymerization efficiency and degree of conversion [50,80]; however, degree of conversion was not measured in the present study, and this relationship should therefore be considered a possible explanation rather than a direct mechanistic conclusion.

Similarly, Fráter et al. [81] demonstrated that different light-curing methods significantly affected the microhardness of resin-based restorative materials, with light transmission through a glass fiber post improving the hardness of short fiber-reinforced composites in deeper regions of the root canal. Although the materials, specimen geometry, and curing conditions differed from those used in the present study, these findings further emphasize the importance of light delivery conditions for the mechanical response of light-cured restorative materials.

Twinky Star^®^ Silver showed one of the most favorable mechanical profiles among the colored compomers, with relatively high hardness and indentation modulus values at both curing times. The mean values were generally similar or slightly higher after 40 s curing, indicating comparatively stable mechanical behavior under the investigated conditions. Charisma A2 showed a significant curing-time-related change in mechanical response: after 40 s curing, HV, HM, HIT, and EIT were significantly higher, while residual indentation depth was significantly lower than after 20 s curing. Charisma A2 cured for 40 s also exhibited the highest HIT value among the investigated groups.

The Boston A3 results showed condition dependent differences in several indentation parameters. Welch’s ANOVA indicated significant differences in HV, HM, HIT, and EIT across the investigated conditions. The 7 mm group showed markedly lower mean hardness and modulus values together with substantially greater variability; however, pairwise comparisons involving this condition did not reach statistical significance. Therefore, the 7 mm results should be interpreted cautiously and regarded primarily as evidence of increased variability under the investigated configuration rather than definitive evidence of a distance-related reduction in mechanical properties. Nevertheless, the observed tendency is consistent with previous studies reporting reduced polymerization effectiveness and microhardness as the distance from the curing light increases [82,83].

SEM and stereomicroscopic observations confirmed that the tested materials differed in surface and structural appearance. The Twinky Star^®^ variants showed heterogeneous surfaces with visible dispersed particles embedded in the resin matrix. Their stereomicroscopic appearance reflected their commercial color variants and the presence of reflective decorative particles. Although the general SEM morphology of Twinky Star^®^ materials was broadly similar, EDS analysis revealed differences in elemental composition, especially in filler-related elements. This indicates that color variants may differ not only in pigment content but also in the distribution or relative contribution of inorganic phases.

Boston A3 and Charisma A2 differed from the Twinky Star^®^ materials and from each other. Boston A3 showed a more irregular and cracked surface with large plate-like particles, whereas Charisma A2 exhibited a more homogeneous morphology with numerous fine particles. EDS analysis indicated different filler systems, with Boston A3 showing elements consistent with aluminosilicate and strontium-containing components, while Charisma A2 showed a composition characteristic of silica and barium-containing glass fillers. These structural and compositional differences may contribute to the differences observed between Boston A3, Charisma A2, and the Twinky Star^®^ compomers in the mechanical and surface analyses. Similar relationships between filler composition, morphology, and material performance have been widely reported for resin-based restorative materials [11,52].

Spectrophotometric analysis confirmed significant differences among the Twinky Star^®^ color variants for all investigated CIELAB parameters. Blue was significantly darker than Gold, Lemon, and Silver, whereas Silver showed significantly lower chroma than the other Twinky Star^®^ variants. Significant differences were also observed in hue angle and chromatic coordinates, confirming that the investigated variants represented clearly distinct optical profiles. The relatively high variability observed for Silver, particularly in hue angle, may reflect local optical heterogeneity associated with its reflective decorative particles. Similar shade-dependent differences in optical properties among colored compomers were previously reported by Hwang et al. [74].

Wettability analysis showed that the Twinky Star^®^ variants did not differ significantly in water contact angle, whereas significant differences were observed for the diiodomethane contact angle. Gold showed higher diiodomethane contact angles than Blue and Lemon, while Silver also differed significantly from Blue. The calculated surface free energy parameters were evaluated descriptively. Twinky Star^®^ Blue showed the highest total surface free energy among the colored compomers, whereas Gold exhibited the highest polar ratio. Boston A3, included for comparative purposes, showed the highest water contact angle and the lowest total surface free energy and polar ratio, corresponding to a comparatively hydrophobic and weakly polar surface. Similar differences in surface physicochemical properties have been reported for resin-based dental materials. Szalóki et al. [84] demonstrated that different resin composites exhibit distinct surface free energy values and wetting behavior toward dental adhesives, indicating substantial variability in surface properties among restorative materials. Liber-Kneć and Łagan [61] showed that contact angle and surface free energy measurements are sensitive tools for characterizing dental biomaterials and assessing differences in their surface physicochemical properties. Furthermore, Rüttermann et al. [85] reported that the incorporation of low-surface-tension additives into resin composites significantly increased contact angle and reduced surface free energy, whereas Rüttermann et al. [86] confirmed that these effects persisted after chewing simulation, resulting in more hydrophobic composite surfaces.

The surface free energy results are clinically relevant because wettability influences the interaction of restorative materials with water, saliva, proteins, stains, and biofilm-forming microorganisms. However, these parameters should not be interpreted in isolation. A more wettable surface may interact more readily with the oral environment, but clinical behavior also depends on surface roughness, polishing quality, filler exposure, aging, water sorption, and degradation, all of which may substantially modify surface characteristics over time [87,88]. Therefore, contact angle and surface free energy measurements provide useful baseline information on surface physicochemical character, but they do not alone predict long-term clinical performance.

Taken together, the results indicate that the investigated Twinky Star^®^ color variants differ in several functional characteristics. Lemon exhibited the highest accumulated thermal exposure, Gold showed comparatively lower mechanical performance, Blue showed the lowest thermal response, and Silver exhibited relatively high hardness- and modulus-related values. Because the individual variants may differ not only in pigmentation but also in decorative particles and filler-related composition, these differences cannot be attributed to color alone. Rather, they indicate that shade-associated compositional and optical differences may influence light absorption, thermal response, mechanical behavior, and surface characteristics [22,50].

From a clinical perspective, the findings suggest that shade selection in colored pediatric compomers may have implications beyond appearance. While the attractive color and glitter effect of such materials may improve child acceptance and cooperation, individual color variants may differ in curing-related heat generation and mechanical performance. Extended curing affected selected mechanical parameters, particularly HM and EIT, while also modifying thermal exposure in a material-dependent manner. This balance is especially important in pediatric dentistry, where restorations may be placed in smaller teeth with thinner remaining dentin and potentially greater sensitivity to thermal effects.

This study has several limitations. The experiments were performed under controlled in vitro conditions, which do not fully reproduce the clinical environment. In vivo, temperature rise may be affected by remaining dentin thickness, pulpal blood flow, cavity geometry, moisture, and heat dissipation through surrounding tissues. The surface free energy measurements were performed on prepared specimens under static conditions and did not include aging, brushing simulation, salivary pellicle formation, or biofilm exposure. In addition, SEM/EDS analysis provides local information from selected areas and may not fully represent the entire material volume. Future studies should include degree of conversion analysis, long-term water aging, wear testing, color stability after staining, and biological evaluation of pulp- or biofilm-related responses. The Boston A3 curing-distance dataset showed substantial variability, particularly at 7 mm, which limited the statistical power of pairwise comparisons for this condition.

Furthermore, sectioning and wet polishing performed before instrumented indentation removed the original light-cured surface layer and may have influenced the measured mechanical response. The exact thickness of the removed material was not quantified, and surface roughness was not measured before and after preparation. Therefore, the indentation results should be interpreted as representative of the prepared and polished subsurface rather than of the original as-cured surface. Future studies should quantify material removal and surface roughness before and after specimen preparation to assess their potential influence on indentation measurements.

Overall, the study shows that the investigated Twinky Star^®^ color variants differ in thermal, mechanical, elemental, optical, and surface characteristics. These findings indicate that commercially available color variants of pediatric restorative materials may differ in functional characteristics in addition to their visual appearance. The results support a more comprehensive evaluation of colored compomers, taking into account both their behavioral advantages in pediatric dentistry and their variant-associated material properties.

## 5. Conclusions

The present study demonstrated that the investigated Twinky Star^®^ compomer color variants differed not only in visual appearance but also in thermal, mechanical, elemental, optical, and surface physicochemical characteristics, while ATR-FTIR revealed broadly comparable chemical profiles with variant-dependent differences in band intensity. All investigated materials showed temperature increases during light curing and exceeded the analytical threshold of 40 °C. Among the Twinky Star^®^ variants, Lemon generated the highest accumulated thermal exposure, whereas Blue showed the lowest thermal response. Gold also produced high temperatures, suggesting that shade-associated differences in pigmentation, translucency, decorative particles, and filler-related composition may contribute to differences in light absorption and heat generation.

Mechanical properties differed significantly among the Twinky Star^®^ color variants. Shade had a significant effect on all investigated indentation parameters, whereas curing time significantly affected HM and EIT. No significant shade × curing time interaction was observed. Gold showed comparatively lower hardness- and modulus-related values, whereas Lemon and Silver generally exhibited higher values. Charisma A2 showed significant changes in most indentation parameters after prolonged curing. For Boston A3, the 7 mm condition was characterized by lower mean mechanical parameters and high variability; however, pairwise differences involving this condition were not statistically significant.

SEM and EDS analyses confirmed differences in morphology and elemental composition between the investigated materials. Spectrophotometric analysis demonstrated significant differences among the Twinky Star^®^ color variants, while wettability analysis showed significant differences in the diiodomethane contact angle but not in the water contact angle. Surface free energy parameters showed descriptive differences among the materials. Boston A3 exhibited the highest water contact angle and the lowest total surface free energy and polar ratio, whereas Twinky Star^®^ Blue and Charisma A2 showed the highest total surface free energy values.

ATR-FTIR analysis indicated that the Twinky Star^®^ color variants shared a broadly comparable chemical profile, with similar positions of the main absorption bands. Differences were observed mainly in band intensity, suggesting variation in pigments, decorative particles, and filler-related components rather than major differences in the basic chemical structure. These observations were consistent with SEM/EDS findings showing a broadly similar material structure together with variant dependent differences in selected elemental components.

Overall, the findings indicate that Twinky Star^®^ color variants should not be considered purely aesthetic alternatives. The observed differences cannot be attributed to color alone, but rather may reflect shade-associated differences in pigmentation, decorative particles, optical behavior, and filler-related composition. These factors, together with curing conditions, may influence thermal response, mechanical properties, optical characteristics, and surface behavior. Therefore, curing protocols should be selected with consideration of the specific material and clinical conditions.

## Figures and Tables

**Table 1:** Dental restorative materials included in the study, classified according to material type, manufacturer and shade. The investigated materials comprised four colored Twinky Star^®^ compomer variants and two commonly used tooth-shaded microhybrid resin composites included for comparison.

Product	Type	Shade
Twinky Star^®^ (VOCO GmbH, Cuxhaven, Germany)	Compomer	blue, lemon, gold, silver
Boston^®^ (Arkona, Nasutow, Poland)	Microhybrid composite	A3
Charisma^®^ (Kulzer, Hanau, Germany)	Microhybrid composite	A2

**Table 2:** Temperature characteristics recorded during light-curing of the tested dental materials. Values are presented as mean ± standard deviation. *t*_1_—exposure time; *T*_0_—initial temperature; *t*max—time to reach maximum temperature; *T*max—maximum temperature; *T*_1_—temperature after the exposure period; *t*_40°C_—time during which the temperature exceeded 40 °C; Δ*T*—temperature increase relative to the initial temperature; *A*_40_—area under the temperature–time curve above 40 °C.

Material	*t*_1_ (s)	*T*_0_ (°C)	*t*_max_ (s)	*T*_max_ (°C)	*T*_1_ (°C)	*t*_40°C_ (s)	Δ*T* (°C)	*A*_40_ (s·°C)
TS Blue	20	22.6 ± 0.1 ^a^	4.3 ± 0.9 ^b^	41.9 ± 0.2 ^c^	37.8 ± 0.1 ^e^	6.8 ± 0.5 ^e^	19.6 ± 0.1 ^c^	328 ± 19 ^f^
TS Blue	40	22.5 ± 0.1 ^a^	23.9 ± 20 ^a^	46.2 ± 1.0 ^b^	40.2 ± 2.5 ^d^	21.9 ± 16.8 ^d^	23.6 ± 10 ^c^	1024 ± 33 ^e^
TS Gold	20	22.5 ± 0.1 ^a^	20.2 ± 0.1 ^a^	69.7 ± 1.3 ^a^	69.7 ± 1.3 ^a^	33.9 ± 1.5 ^c^	47.2 ± 1.3 ^a^	1991 ± 28 ^c^
TS Gold	40	22.5 ± 0.1 ^a^	30.2 ± 15.5 ^a^	57.8 ± 1.2 ^b^	56.8 ± 2.6 ^c^	51.1 ± 0.3 ^b^	35.3 ± 1.2 ^b^	2779 ± 84 ^b^
TS Lemon	20	22.5 ± 0.1 ^a^	20.2 ± 0.1 ^a^	57.6 ± 7.7 ^b^	57.6 ± 7.7 ^c^	28.1 ± 3.3 ^c^	35.2 ± 7.7 ^a^	1487 ± 315 ^d^
TS Lemon	40	22.5 ± 0.5 ^a^	41.1 ± 2.8 ^a^	66.6 ± 6.5 ^a^	66.5 ± 6.8 ^b^	62.5 ± 3.6 ^a^	44.1 ± 6.6 ^a^	3648 ± 123 ^a^
TS Silver	20	22.6 ± 0.5 ^a^	5.3 ± 0.1 ^b^	52.3 ± 2.9 ^b^	45.8 ± 3.5 ^d^	22.9 ± 2.1 ^d^	29.7 ± 2.9 ^b^	1141 ± 151 ^e^
TS Silver	40	22.5 ± 0.4 ^a^	31.8 ± 0.1 ^a^	57.4 ± 1.2 ^a^	54.3 ± 0.2 ^b^	50.5 ± 3.1 ^b^	36.5 ± 2.5 ^b^	2644 ± 349 ^a^

Post hoc test results (Tukey’s HSD test) for the tested material groups (*n* = 8). Values sharing the same superscript letter within a given column are not statistically significantly different (*p* > 0.05).

**Table 3:** Temperature characteristics recorded during light-curing of Boston A3 and Charisma A2 dental materials.

Material	*t*_1_ (s)	*T*_0_ (°C)	*t*_max_ (s)	*T*_max_ (°C)	*T*_1_ (°C)	*t*_40°C_ (s)	Δ*T* (°C)	*A*_40_ (s·°C)
Boston A3	20	22.5 ± 0.1 ^a^	10.6 ± 0.2 ^a^	48.6 ± 4.4 ^b^	46.4 ± 6.2 ^b^	20.8 ± 12.4 ^b^	26.1 ± 6.0 ^a^	1015 ± 25 ^a^
Boston A3	40	22.5 ± 0.1 ^a^	10.6 ± 0.2 ^a^	48.1 ± 4.3 ^a^	44.3 ± 6.3 ^b^	40.1 ± 16.9 ^a^	25.6 ± 6.1 ^a^	1856 ± 62 ^a^
Charisma A2	20	22.5 ± 0.1 ^a^	6.6 ± 0.1 ^b^	46.4 ± 4.2 ^a^	44.7 ± 6.2 ^b^	20.8 ± 12.3 ^b^	23.9 ± 6.0 ^a^	980 ± 24 ^a^
Charisma A2	40	22.5 ± 0.1 ^a^	9.3 ± 0.1 ^a^	49.7 ± 4.5 ^a^	47.4 ± 6.4 ^a^	46.1 ± 17.2 ^a^	27.2 ± 6.5 ^a^	2208 ± 51 ^a^

Post hoc test results (Tukey’s HSD test) for the tested material groups (with *n* = 8 replicates). Values sharing the same superscript letter within a given column are not statistically significantly different (*p* > 0.05).

**Table 4:** Two-way ANOVA results for the effects of material type and curing time on maximum polymerization temperature (*T*max).

Effect	SS	df	MS	F	*p*-Value	Partial η^2^
Intercept	274,991	1	274,991	22,740	<0.001	0.996
Material	5213	5	1043	86.20	<0.001	0.837
Curing time	59	1	59	4.88	0.023	0.055
Material × Curing time	1056	5	211	17.47	<0.001	0.510
Error	1016	84	12			

**Table 5:** Spectrophotometric color parameters of the investigated dental materials expressed in the CIELAB color space. Values are presented as mean ± SD and include lightness (L*), chroma (C*), hue angle (h°), and chromatic coordinates (a* and b*), describing differences in lightness, color saturation, hue, and color direction among the tested Twinky Star^®^, Boston A3, and Charisma A2 materials. Different superscript letters within the same column indicate statistically significant differences among the Twinky Star^®^ color variants (Welch’s one-way ANOVA followed by the Games–Howell post hoc test, *p* < 0.05). Boston A3 and Charisma A2 were included as commonly used tooth-shaded resin composites for descriptive comparison and were not included in the inferential statistical analysis.

Material	L*	C*	h°	a*	b*
Twinky Star Blue	39.43 ± 5.62 ^b^	36.68 ± 16.59 ^a^	276.80 ± 2.60 ^a^	5.60 ± 4.13 ^ab^	−39.20 ± 17.20 ^c^
Twinky Star Gold	59.00 ± 5.68 ^a^	40.67 ± 10.78 ^a^	83.00 ± 2.76 ^b^	4.20 ± 0.75 ^b^	43.17 ± 7.93 ^a^
Twinky Star Lemon	55.77 ± 7.56 ^a^	31.47 ± 9.40 ^a^	97.63 ± 5.97 ^c^	−5.22 ± 5.18 ^c^	31.45 ± 9.01 ^a^
Twinky Star Silver	65.70 ± 8.91 ^a^	5.15 ± 3.51 ^b^	134.78 ± 58.16 ^bc^	−1.10 ± 1.13 ^ac^	4.13 ± 4.72 ^b^
Boston A3	83.25 ± 0.21	6.95 ± 0.35	75.70 ± 5.37	1.70 ± 0.00	8.00 ± 0.14
Charisma A2	58.90 ± 2.40	4.30 ± 0.71	128.45 ± 1.34	−2.65 ± 0.35	3.35 ± 0.64

**Table 6:** Water (θ1) and diiodomethane (θ2) contact angles and surface free energy parameters of the tested dental materials. Values are presented as mean ± SD. The dispersive component (γs^d^), polar component (γs^p^), total surface free energy (γs), and polar ratio were calculated using the Owens–Wendt method. Different superscript letters within the same contact-angle column indicate statistically significant differences among the Twinky Star^®^ color variants (Welch’s one-way ANOVA followed by the Games–Howell post hoc test, *p* < 0.05). Boston A3 and Charisma A2 were included for descriptive comparison and were not included in the inferential statistical analysis. For Twinky Star^®^ Silver, two outlying water contact angle values above 100° were excluded prior to calculation.

Material	Contact Angle [°]		Surface Energy [mJ/m^2^]			Polar Ratio [%]
	θ_1_ [°]	θ_2_ [°]	γs^d^	γs^p^	γs	
Twinky Star Blue	63.44 ± 7.79 ^a^	28.39 ± 1.79 ^c^	44.88 ± 0.71	8.98 ± 3.38	53.85 ± 3.40	16.67 ± 5.31
Twinky Star Gold	62.72 ± 3.80 ^a^	44.16 ± 3.82 ^a^	37.46 ± 2.01	11.78 ± 2.09	49.23 ± 2.31	23.92 ± 3.64
Twinky Star Lemon	63.99 ± 7.33 ^a^	34.48 ± 7.55 ^bc^	42.27 ± 3.47	9.50 ± 3.84	51.76 ± 4.33	18.34 ± 5.92
Twinky Star Silver	61.23 ± 2.48 ^a^	38.79 ± 7.28 ^ab^	40.21 ± 3.71	11.59 ± 1.78	51.80 ± 2.53	22.37 ± 4.02
Boston A3	86.93 ± 1.33	52.05 ± 0.37	33.12 ± 0.21	2.58 ± 0.34	35.71 ± 0.37	7.24 ± 0.89
Charisma A2	62.53 ± 0.72	31.28 ± 2.10	43.69 ± 0.90	9.78 ± 0.40	53.47 ± 0.64	18.29 ± 0.80

**Table 7:** Instrumented indentation parameters of the Twinky Star^®^ materials presented as mean ± SD.

Material	h_r_ [µm]	HV	HM [N/mm^2^]	H_IT_ [N/mm^2^]	E_IT_ [kN/mm^2^]	η_IT_ [%]
Twinky Star blue (20 s)	4.34 ± 0.18	74.24 ± 5.27	518.86 ± 56.32	786.51 ± 55.88	13.75 ± 2.74	31.46 ± 4.89
Twinky Star blue (40 s)	4.22 ± 0.16	77.01 ± 5.43	591.43 ± 23.32	815.95 ± 57.57	17.29 ± 1.01	36.86 ± 1.24
Twinky Star gold (20 s)	4.86 ± 0.25	59.58 ± 6.21	438.24 ± 26.51	631.24 ± 65.82	12.67 ± 1.63	29.87 ± 3.90
Twinky Star gold (40 s)	4.54 ± 0.42	67.43 ± 10.67	514.41 ± 52.05	714.44 ± 113.01	15.38 ± 1.70	32.89 ± 4.34
Twinky Star lemon (20 s)	4.12 ± 0.09	79.46 ± 2.65	593.16 ± 13.63	841.90 ± 28.06	15.94 ± 1.07	37.74 ± 1.51
Twinky Star lemon (40 s)	4.14 ± 0.21	82.34 ± 4.34	628.27 ± 40.21	851.22 ± 78.71	18.58 ± 1.06	34.62 ± 3.22
Twinky Star Silver (20 s)	4.14 ± 0.13	79.37 ± 4.82	602.05 ± 35.16	840.95 ± 51.11	16.90 ± 1.22	36.00 ± 1.69
Twinky Star Silver (40 s)	4.12 ± 0.12	80.54 ± 3.83	619.80 ± 11.38	853.35 ± 40.53	18.30 ± 1.40	36.06 ± 1.73

**Table 8:** Instrumented indentation parameters of the composite materials presented as mean ± SD.

Material	h_r_ [µm]	HV	HM [N/mm^2^]	H_IT_ [N/mm^2^]	E_IT_ [kN/mm^2^]	η_IT_ [%]
Charisma A2 (20 s)	4.26 ± 0.05	74.45 ± 1.43	560.36 ± 6.12	788.79 ± 15.12	15.36 ± 0.20	36.52 ± 0.34
Charisma A2 (40 s)	4.05 ± 0.09	83.27 ± 3.43	641.27 ± 27.08	882.24 ± 36.34	18.85 ± 1.25	35.63 ± 0.76
Boston A3 (20 s)	4.28 ± 0.04	74.73 ± 1.13	576.20 ± 5.74	791.79 ± 11.95	16.99 ± 0.21	34.71 ± 0.37
Boston A3 2.5 mm	4.46 ± 0.07	68.76 ± 1.94	528.07 ± 11.84	728.47 ± 20.59	15.40 ± 0.31	35.70 ± 0.29
Boston A3 7 mm	6.30 ± 3.65	55.58 ± 36.72	423.57 ± 268.37	588.87 ± 389.00	12.59 ± 5.89	38.81 ± 2.66

**Table 9:** Two-way ANOVA results for the effects of shade and curing time on the instrumented indentation parameters of Twinky Star^®^ materials.

Parameter	Effect	F	df	*p*-Value	Partial η^2^
hr	Shade	10.78	3, 24	<0.001	0.574
	Curing time	1.16	1, 24	0.291	0.046
	Shade × Curing time	1.25	3, 24	0.313	0.135
HV	Shade	12.15	3, 24	<0.001	0.603
	Curing time	0.89	1, 24	0.354	0.036
	Shade × Curing time	1.13	3, 24	0.356	0.124
HM	Shade	18.20	3, 24	<0.001	0.695
	Curing time	6.84	1, 24	0.015	0.222
	Shade × Curing time	1.15	3, 24	0.350	0.126
HIT	Shade	12.15	3, 24	<0.001	0.603
	Curing time	0.89	1, 24	0.354	0.036
	Shade × Curing time	1.13	3, 24	0.356	0.124
EIT	Shade	7.07	3, 24	0.001	0.469
	Curing time	13.31	1, 24	0.001	0.357
	Shade × Curing time	0.24	3, 24	0.868	0.029
ηIT	Shade	3.52	3, 24	0.030	0.306
	Curing time	0.82	1, 24	0.375	0.033
	Shade × Curing time	1.70	3, 24	0.194	0.175

## Data Availability

The original contributions presented in this study are included in the article. Further inquiries can be directed to the corresponding authors.
